# 4,6-Dimeth­oxy-2-(methyl­sulfan­yl)pyrimidine–4-hy­droxy­benzoic acid (1/1)

**DOI:** 10.1107/S1600536812046338

**Published:** 2012-11-24

**Authors:** Kaliyaperumal Thanigaimani, Abbas Farhadikoutenaei, Suhana Arshad, Ibrahim Abdul Razak, Kasthuri Balasubramani

**Affiliations:** a School of Physics, Universiti Sains Malaysia, 11800 USM, Penang, Malaysia; b Department of Physics, Faculty of Science, University of Mazandaran, Babolsar, Iran; c Department of Chemistry, Government Arts College, Thonthonimalai, Karur, Tamil Nadu, India

## Abstract

The base mol­ecule of the title co-crystal, C_7_H_10_N_2_O_2_S·C_7_H_6_O_3_, is essentially planar, with a maximum deviation of 0.0806 (14) Å for all non-H atoms. The acid mol­ecule is also nearly planar, with a dihedral angle of 8.12 (14)° between the benzene ring and the carb­oxy group. In the crystal, the acid mol­ecules form an inversion dimer through a pair of O—H⋯O hydrogen bonds with an *R*_2_^2^(8) ring motif. The pyrimidine mol­ecules are linked on both sides of the dimer into a heterotetra­mer *via* O—H⋯N and C—H⋯O hydrogen bonds with *R*_2_^2^(8) ring motifs. The heterotetra­mers are further linked by weak C—H⋯O hydrogen bonds, forming a tape structure along [1-10].

## Related literature

For general background to substituted pyrimidines, see: Hunt *et al.* (1980 ▶); Baker & Santi (1965 ▶); Holy *et al.* (1974 ▶). For 4-hy­droxy­benzoic acid, see: Vishweshwar *et al.* (2003 ▶). For related structures, see: Balasubramani & Fun (2009 ▶); Hemamalini & Fun (2010 ▶). For hydrogen-bond motifs, see: Bernstein *et al.* (1995 ▶). For bond-length data, see: Allen *et al.* (1987 ▶). For the stability of the temperature controller used for the data collection, see: Cosier & Glazer (1986 ▶).
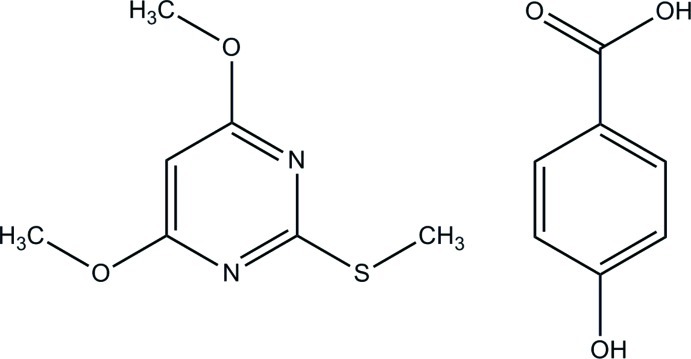


## Experimental

### 

#### Crystal data

C_7_H_10_N_2_O_2_S·C_7_H_6_O_3_
*M* *_r_* = 324.35
Triclinic, 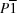

*a* = 6.9923 (5) Å
*b* = 10.2887 (8) Å
*c* = 10.7578 (8) Å
α = 77.419 (2)°
β = 83.381 (2)°
γ = 89.209 (2)°
*V* = 750.27 (10) Å^3^
*Z* = 2
Mo *K*α radiation
μ = 0.24 mm^−1^
*T* = 100 K
0.44 × 0.37 × 0.23 mm

#### Data collection

Bruker SMART APEXII CCD area-detector diffractometer
Absorption correction: multi-scan (*SADABS*; Bruker, 2009 ▶) *T*_min_ = 0.901, *T*_max_ = 0.947
13682 measured reflections
3393 independent reflections
3105 reflections with *I* > 2σ(*I*)
*R*_int_ = 0.024

#### Refinement

*R*[*F* ^2^ > 2σ(*F* ^2^)] = 0.032
*wR*(*F* ^2^) = 0.093
*S* = 1.10
3393 reflections
210 parameters
1 restraint
H atoms treated by a mixture of independent and constrained refinement
Δρ_max_ = 0.46 e Å^−3^
Δρ_min_ = −0.27 e Å^−3^

### 

Data collection: *APEX2* (Bruker, 2009 ▶); cell refinement: *SAINT* (Bruker, 2009 ▶); data reduction: *SAINT*; program(s) used to solve structure: *SHELXTL* (Sheldrick, 2008 ▶); program(s) used to refine structure: *SHELXTL*; molecular graphics: *SHELXTL*; software used to prepare material for publication: *SHELXTL* and *PLATON* (Spek, 2009 ▶).

## Supplementary Material

Crystal structure: contains datablock(s) global, I. DOI: 10.1107/S1600536812046338/is5216sup1.cif

Structure factors: contains datablock(s) I. DOI: 10.1107/S1600536812046338/is5216Isup2.hkl

Supplementary material file. DOI: 10.1107/S1600536812046338/is5216Isup3.cml

Additional supplementary materials:  crystallographic information; 3D view; checkCIF report

## Figures and Tables

**Table 1:** Hydrogen-bond geometry (Å, °)

*D*—H⋯*A*	*D*—H	H⋯*A*	*D*⋯*A*	*D*—H⋯*A*
O4—H1*O*4⋯O3^i^	0.86 (2)	1.76 (2)	2.6189 (14)	172 (3)
O5—H1*O*5⋯N1^ii^	0.80 (3)	1.99 (3)	2.7562 (14)	162 (3)
C9—H9*A*⋯O1^ii^	0.95	2.44	3.3437 (16)	160
C12—H12*A*⋯O2^iii^	0.95	2.59	3.3340 (16)	135

Symmetry codes: (i) 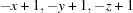; (ii) 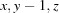; (iii) 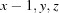.

**Fig. 1.**
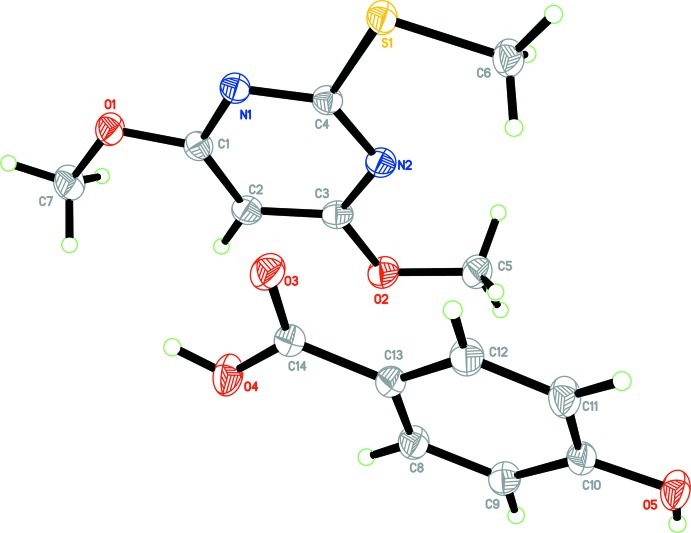
The molecular structure of the title compound with atom labels with 50% probability displacement ellipsoids.

**Fig. 2.**
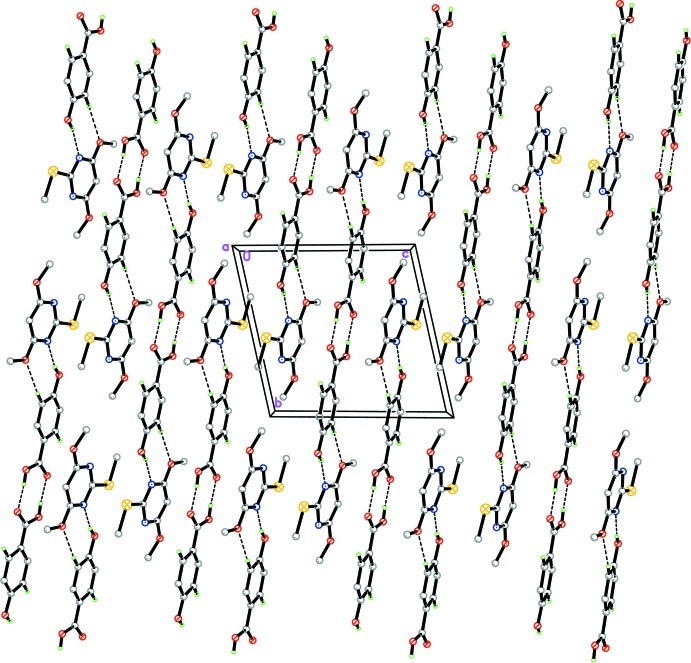
The crystal packing of the title compound. The H atoms not involved in the intermolecular interactions (dashed lines) have been omitted for clarity.

## supplementary crystallographic information

### Comment

Pyrimidine and aminopyrimidine derivatives are biologically important compounds as they occur in nature as components of nucleic acids. Some aminopyrimidine derivatives are used as antifolate drugs (Hunt *et al.*, 1980; Baker & Santi, 1965). 2-Thiopyrimidine shows a strong bacteriostatic activity in *vitro* on *E. coli* (Holy *et al.*, 1974). 4-Hydroxybenzoic acid is a good hydrogen-bond donor and can form co-crystals with other organic molecules (Vishweshwar *et al.*, 2003). We have recently reported the crystal structures of 4,6-dimethoxy-2-(methylsulfanyl)pyrimidine (Balasubramani & Fun, 2009) and 4,6-dimethoxy-2-(methylsulfanyl)pyrimidinium chloride (Hemamalini & Fun, 2010). In order to study some potential hydrogen bonding interactions the crystal structure determination of the title compound (I) was carried out.

The asymmetric unit (Fig. 1), contains one 4,6-dimethoxy-2-(methylsulfanyl)pyrimidine molecule and one 4-hydroxybenzoic acid molecule. The 4,6-dimethoxy-2-(methylsulfanyl)pyrimidine molecule is planar, with a maximum deviation of 0.0806 (14) Å of atom C5 from a mean plane of all non-H atoms. The carboxy group of the 4-hydroxybenzoic acid molecule is slightly twisted from the attached ring by 8.12 (14)°. The bond lengths (Allen *et al.*, 1987) and angles are normal.

In the crystal packing (Fig. 2), the 4-hydroxybenzoic acid molecules are centrosymmetrically paired through a pair of O4—H1O4···O3^i^ hydrogen bonds (symmetry code in Table 1) to form an *R*_2_^2^(8) (Bernstein *et al.*, 1995) ring motif. In addition, the 4,6-dimethoxy-2-methylthiopyrimidine molecule and 4-hydroxybenzoic acid molecule are linked *via* intermolecular O5—H1O5···N1^ii^ and C9—H9A···O1^ii^ hydrogen bonds (symmetry code in Table 1), generating *R*_2_^2^(8) ring motifs. The crystal structure is further stabilized by weak C12—H12A···O2^iii^ hydrogen bonds (symmetry code in Table 1), forming a supramolecular tape.

### Experimental

Hot methanol solutions (20 ml) of 4,6-Dimethoxy-2-methylthiopyrimidine (46 mg, Aldrich) and 4-hydroxybenzoic acid (39 mg, Merck) were mixed and warmed over a heating magnetic stirrer hotplate for a few minutes. The resulting solution was allowed to cool slowly at room temperature and single crystals of the title compound (I) appeared after a few days.

### Refinement

O-bound H atoms were located in a difference Fourier maps. Atom H1O5 was refined freely, while atom H1O4 was refined with a bond length restraint O—H = 0.85 (1) Å [refined distances: O5—H1O5 = 0.79 (3) Å and O4—H1O4 = 0.862 (10) Å]. The remaining H atoms were positioned geometrically (C—H = 0.95 and 0.98 Å) and were refined using a riding model, with *U*_iso_(H) = 1.2*U*_eq_(C) or 1.5*U*_eq_(methyl C). A rotating-group model was used for the methyl group. In the final refinement, five outliers were omitted (-2 -6 2, -3 6 3, 4 -5 1, -3 5 1 and 3 6 0).

### Crystal data

**Table:**

C_7_H_10_N_2_O_2_S·C_7_H_6_O_3_	*Z* = 2
*M**_r_* = 324.35	*F*(000) = 340
Triclinic, *P*1	*D*_x_ = 1.436 Mg m^−^^3^
Hall symbol: -P 1	Mo *K*α radiation, λ = 0.71073 Å
*a* = 6.9923 (5) Å	Cell parameters from 9952 reflections
*b* = 10.2887 (8) Å	θ = 2.5–32.6°
*c* = 10.7578 (8) Å	µ = 0.24 mm^−^^1^
α = 77.419 (2)°	*T* = 100 K
β = 83.381 (2)°	Block, colourless
γ = 89.209 (2)°	0.44 × 0.37 × 0.23 mm
*V* = 750.27 (10) Å^3^	

### Data collection

**Table:**

Bruker SMART APEXII CCD area-detector diffractometer	3393 independent reflections
Radiation source: fine-focus sealed tube	3105 reflections with *I* > 2σ(*I*)
Graphite monochromator	*R*_int_ = 0.024
φ and ω scans	θ_max_ = 27.5°, θ_min_ = 2.0°
Absorption correction: multi-scan (*SADABS*; Bruker, 2009)	*h* = −9→9
*T*_min_ = 0.901, *T*_max_ = 0.947	*k* = −13→13
13682 measured reflections	*l* = −13→13

### Refinement

**Table:**

Refinement on *F*^2^	Primary atom site location: structure-invariant direct methods
Least-squares matrix: full	Secondary atom site location: difference Fourier map
*R*[*F*^2^ > 2σ(*F*^2^)] = 0.032	Hydrogen site location: inferred from neighbouring sites
*wR*(*F*^2^) = 0.093	H atoms treated by a mixture of independent and constrained refinement
*S* = 1.10	*w* = 1/[σ^2^(*F*_o_^2^) + (0.0508*P*)^2^ + 0.2589*P*] where *P* = (*F*_o_^2^ + 2*F*_c_^2^)/3
3393 reflections	(Δ/σ)_max_ = 0.001
210 parameters	Δρ_max_ = 0.46 e Å^−^^3^
1 restraint	Δρ_min_ = −0.27 e Å^−^^3^

### Special details

**Table:**

Experimental. The crystal was placed in the cold stream of an Oxford Cryosystems Cobra open-flow nitrogen cryostat (Cosier & Glazer, 1986) operating at 100.0 (1) K.
Geometry. All e.s.d.'s (except the e.s.d. in the dihedral angle between two l.s. planes) are estimated using the full covariance matrix. The cell e.s.d.'s are taken into account individually in the estimation of e.s.d.'s in distances, angles and torsion angles; correlations between e.s.d.'s in cell parameters are only used when they are defined by crystal symmetry. An approximate (isotropic) treatment of cell e.s.d.'s is used for estimating e.s.d.'s involving l.s. planes.
Refinement. Refinement of *F*^2^ against ALL reflections. The weighted *R*-factor *wR* and goodness of fit *S* are based on *F*^2^, conventional *R*-factors *R* are based on *F*, with *F* set to zero for negative *F*^2^. The threshold expression of *F*^2^ > σ(*F*^2^) is used only for calculating *R*-factors(gt) *etc*. and is not relevant to the choice of reflections for refinement. *R*-factors based on *F*^2^ are statistically about twice as large as those based on *F*, and *R*- factors based on ALL data will be even larger.

### Fractional atomic coordinates and isotropic or equivalent isotropic displacement parameters (Å^2^)

**Table:**

	*x*	*y*	*z*	*U*_iso_*/*U*_eq_	
S1	0.22119 (4)	0.45956 (3)	0.93029 (3)	0.02129 (10)	
O1	0.78663 (12)	0.67424 (9)	0.67045 (9)	0.0237 (2)	
O2	0.81296 (13)	0.20863 (9)	0.83750 (9)	0.0261 (2)	
O3	0.31596 (13)	0.39971 (9)	0.58570 (9)	0.0251 (2)	
O4	0.61173 (13)	0.34673 (10)	0.50895 (9)	0.0272 (2)	
O5	0.28845 (14)	−0.22653 (9)	0.78692 (9)	0.0260 (2)	
N1	0.53552 (14)	0.56189 (10)	0.79118 (10)	0.0195 (2)	
N2	0.54001 (14)	0.32727 (10)	0.87876 (10)	0.0199 (2)	
C1	0.71747 (17)	0.55457 (12)	0.73576 (11)	0.0197 (2)	
C2	0.81861 (17)	0.43673 (13)	0.74714 (12)	0.0214 (2)	
H2A	0.9460	0.4324	0.7067	0.026*	
C3	0.71973 (17)	0.32502 (13)	0.82225 (11)	0.0207 (2)	
C4	0.45790 (16)	0.44686 (12)	0.85972 (11)	0.0185 (2)	
C5	0.7153 (2)	0.09621 (13)	0.92394 (13)	0.0300 (3)	
H5A	0.8014	0.0196	0.9344	0.045*	
H5B	0.5998	0.0741	0.8888	0.045*	
H5C	0.6784	0.1185	1.0074	0.045*	
C6	0.16623 (18)	0.28891 (13)	1.00996 (13)	0.0261 (3)	
H6A	0.0352	0.2828	1.0546	0.039*	
H6B	0.2579	0.2581	1.0721	0.039*	
H6C	0.1756	0.2331	0.9464	0.039*	
C7	0.98129 (18)	0.67934 (14)	0.60767 (13)	0.0271 (3)	
H7A	1.0168	0.7718	0.5665	0.041*	
H7B	0.9897	0.6253	0.5427	0.041*	
H7C	1.0694	0.6444	0.6712	0.041*	
C8	0.55397 (17)	0.07913 (13)	0.62423 (12)	0.0219 (2)	
H8A	0.6798	0.1082	0.5862	0.026*	
C9	0.51922 (17)	−0.05487 (13)	0.67699 (12)	0.0220 (2)	
H9A	0.6210	−0.1170	0.6762	0.026*	
C10	0.33342 (18)	−0.09825 (12)	0.73148 (11)	0.0213 (2)	
C11	0.18445 (18)	−0.00551 (13)	0.73189 (13)	0.0266 (3)	
H11A	0.0576	−0.0348	0.7674	0.032*	
C12	0.22154 (18)	0.12821 (13)	0.68095 (12)	0.0248 (3)	
H12A	0.1205	0.1906	0.6833	0.030*	
C13	0.40666 (17)	0.17230 (12)	0.62604 (11)	0.0199 (2)	
C14	0.44409 (17)	0.31494 (12)	0.57134 (11)	0.0201 (2)	
H1O4	0.625 (4)	0.4317 (11)	0.482 (3)	0.095 (10)*	
H1O5	0.377 (3)	−0.275 (3)	0.781 (2)	0.063 (7)*	

### Atomic displacement parameters (Å^2^)

**Table:**

	*U*^11^	*U*^22^	*U*^33^	*U*^12^	*U*^13^	*U*^23^
S1	0.01540 (15)	0.01880 (17)	0.02680 (17)	0.00153 (11)	0.00292 (11)	−0.00160 (12)
O1	0.0193 (4)	0.0201 (4)	0.0286 (4)	−0.0013 (3)	0.0057 (3)	−0.0024 (4)
O2	0.0238 (4)	0.0198 (5)	0.0323 (5)	0.0059 (4)	0.0029 (4)	−0.0040 (4)
O3	0.0258 (4)	0.0179 (4)	0.0298 (5)	0.0025 (3)	0.0014 (4)	−0.0040 (4)
O4	0.0218 (4)	0.0204 (5)	0.0345 (5)	−0.0021 (4)	0.0034 (4)	0.0010 (4)
O5	0.0235 (5)	0.0152 (4)	0.0350 (5)	0.0010 (4)	0.0047 (4)	−0.0008 (4)
N1	0.0164 (4)	0.0196 (5)	0.0216 (5)	0.0012 (4)	0.0002 (4)	−0.0037 (4)
N2	0.0174 (5)	0.0196 (5)	0.0223 (5)	0.0013 (4)	−0.0004 (4)	−0.0049 (4)
C1	0.0178 (5)	0.0205 (6)	0.0203 (5)	−0.0006 (4)	−0.0001 (4)	−0.0045 (4)
C2	0.0169 (5)	0.0228 (6)	0.0240 (6)	0.0019 (4)	0.0014 (4)	−0.0061 (5)
C3	0.0188 (5)	0.0209 (6)	0.0228 (5)	0.0039 (4)	−0.0019 (4)	−0.0065 (5)
C4	0.0166 (5)	0.0198 (6)	0.0193 (5)	0.0004 (4)	−0.0015 (4)	−0.0050 (4)
C5	0.0351 (7)	0.0196 (6)	0.0313 (7)	0.0053 (5)	0.0039 (5)	−0.0011 (5)
C6	0.0207 (6)	0.0216 (6)	0.0325 (6)	−0.0009 (5)	0.0041 (5)	−0.0019 (5)
C7	0.0185 (6)	0.0287 (7)	0.0308 (7)	−0.0037 (5)	0.0054 (5)	−0.0032 (5)
C8	0.0193 (5)	0.0202 (6)	0.0247 (6)	0.0005 (5)	0.0009 (4)	−0.0037 (5)
C9	0.0199 (6)	0.0195 (6)	0.0254 (6)	0.0031 (4)	−0.0002 (4)	−0.0037 (5)
C10	0.0237 (6)	0.0173 (6)	0.0212 (5)	0.0002 (5)	0.0009 (4)	−0.0023 (4)
C11	0.0211 (6)	0.0210 (6)	0.0331 (7)	0.0007 (5)	0.0070 (5)	−0.0012 (5)
C12	0.0228 (6)	0.0198 (6)	0.0288 (6)	0.0037 (5)	0.0043 (5)	−0.0027 (5)
C13	0.0221 (6)	0.0169 (6)	0.0197 (5)	0.0006 (4)	0.0007 (4)	−0.0034 (4)
C14	0.0217 (6)	0.0185 (6)	0.0195 (5)	0.0004 (4)	−0.0012 (4)	−0.0037 (4)

### Geometric parameters (Å, º)

**Table:**

S1—C4	1.7552 (12)	C5—H5B	0.9800
S1—C6	1.8034 (14)	C5—H5C	0.9800
O1—C1	1.3434 (15)	C6—H6A	0.9800
O1—C7	1.4444 (14)	C6—H6B	0.9800
O2—C3	1.3429 (15)	C6—H6C	0.9800
O2—C5	1.4406 (16)	C7—H7A	0.9800
O3—C14	1.2619 (15)	C7—H7B	0.9800
O4—C14	1.2904 (15)	C7—H7C	0.9800
O4—H1O4	0.862 (10)	C8—C9	1.3851 (17)
O5—C10	1.3497 (15)	C8—C13	1.3987 (17)
O5—H1O5	0.79 (3)	C8—H8A	0.9500
N1—C4	1.3358 (16)	C9—C10	1.3990 (17)
N1—C1	1.3497 (15)	C9—H9A	0.9500
N2—C3	1.3336 (15)	C10—C11	1.4032 (17)
N2—C4	1.3354 (16)	C11—C12	1.3816 (18)
C1—C2	1.3845 (17)	C11—H11A	0.9500
C2—C3	1.3930 (17)	C12—C13	1.3977 (17)
C2—H2A	0.9500	C12—H12A	0.9500
C5—H5A	0.9800	C13—C14	1.4722 (17)
			
C4—S1—C6	101.55 (6)	H6A—C6—H6C	109.5
C1—O1—C7	117.10 (10)	H6B—C6—H6C	109.5
C3—O2—C5	116.61 (10)	O1—C7—H7A	109.5
C14—O4—H1O4	112 (2)	O1—C7—H7B	109.5
C10—O5—H1O5	112.7 (18)	H7A—C7—H7B	109.5
C4—N1—C1	115.43 (10)	O1—C7—H7C	109.5
C3—N2—C4	115.10 (11)	H7A—C7—H7C	109.5
O1—C1—N1	111.95 (10)	H7B—C7—H7C	109.5
O1—C1—C2	124.93 (11)	C9—C8—C13	121.05 (11)
N1—C1—C2	123.11 (11)	C9—C8—H8A	119.5
C1—C2—C3	115.03 (11)	C13—C8—H8A	119.5
C1—C2—H2A	122.5	C8—C9—C10	119.61 (11)
C3—C2—H2A	122.5	C8—C9—H9A	120.2
N2—C3—O2	118.77 (11)	C10—C9—H9A	120.2
N2—C3—C2	124.02 (11)	O5—C10—C9	123.27 (11)
O2—C3—C2	117.20 (11)	O5—C10—C11	117.12 (11)
N2—C4—N1	127.30 (11)	C9—C10—C11	119.60 (11)
N2—C4—S1	118.35 (9)	C12—C11—C10	120.27 (12)
N1—C4—S1	114.35 (9)	C12—C11—H11A	119.9
O2—C5—H5A	109.5	C10—C11—H11A	119.9
O2—C5—H5B	109.5	C11—C12—C13	120.47 (12)
H5A—C5—H5B	109.5	C11—C12—H12A	119.8
O2—C5—H5C	109.5	C13—C12—H12A	119.8
H5A—C5—H5C	109.5	C12—C13—C8	118.99 (11)
H5B—C5—H5C	109.5	C12—C13—C14	119.95 (11)
S1—C6—H6A	109.5	C8—C13—C14	121.05 (11)
S1—C6—H6B	109.5	O3—C14—O4	122.92 (12)
H6A—C6—H6B	109.5	O3—C14—C13	120.49 (11)
S1—C6—H6C	109.5	O4—C14—C13	116.60 (11)
			
C7—O1—C1—N1	179.48 (10)	C6—S1—C4—N2	−0.74 (11)
C7—O1—C1—C2	0.01 (18)	C6—S1—C4—N1	179.09 (9)
C4—N1—C1—O1	−178.96 (10)	C13—C8—C9—C10	0.86 (19)
C4—N1—C1—C2	0.52 (17)	C8—C9—C10—O5	−178.72 (11)
O1—C1—C2—C3	178.46 (11)	C8—C9—C10—C11	0.08 (19)
N1—C1—C2—C3	−0.95 (18)	O5—C10—C11—C12	177.68 (12)
C4—N2—C3—O2	179.53 (10)	C9—C10—C11—C12	−1.2 (2)
C4—N2—C3—C2	−0.63 (17)	C10—C11—C12—C13	1.4 (2)
C5—O2—C3—N2	−5.20 (16)	C11—C12—C13—C8	−0.42 (19)
C5—O2—C3—C2	174.95 (11)	C11—C12—C13—C14	179.62 (12)
C1—C2—C3—N2	1.02 (18)	C9—C8—C13—C12	−0.70 (19)
C1—C2—C3—O2	−179.14 (11)	C9—C8—C13—C14	179.26 (11)
C3—N2—C4—N1	0.13 (18)	C12—C13—C14—O3	7.67 (17)
C3—N2—C4—S1	179.94 (8)	C8—C13—C14—O3	−172.29 (11)
C1—N1—C4—N2	−0.08 (18)	C12—C13—C14—O4	−172.05 (11)
C1—N1—C4—S1	−179.89 (8)	C8—C13—C14—O4	7.99 (17)

### Hydrogen-bond geometry (Å, º)

**Table:**

*D*—H···*A*	*D*—H	H···*A*	*D*···*A*	*D*—H···*A*
O4—H1*O*4···O3^i^	0.86 (2)	1.76 (2)	2.6189 (14)	172 (3)
O5—H1*O*5···N1^ii^	0.80 (3)	1.99 (3)	2.7562 (14)	162 (3)
C9—H9*A*···O1^ii^	0.95	2.44	3.3437 (16)	160
C12—H12*A*···O2^iii^	0.95	2.59	3.3340 (16)	135

Symmetry codes: (i) −*x*+1, −*y*+1, −*z*+1; (ii) *x*, *y*−1, *z*; (iii) *x*−1, *y*, *z*.
